# Examining the association between functional social support, marital status, and memory: a systematic review

**DOI:** 10.1186/s12877-023-03982-3

**Published:** 2023-05-12

**Authors:** Paniz Haghighi, Mark Oremus

**Affiliations:** School of Public Health Sciences, Faculty of Health, University of Waterloo, Waterloo, ON N2L 3G1 Canada

**Keywords:** Social network, Social relationships, Social interaction, Social engagement, Marital status, Cognitive function

## Abstract

**Background:**

Functional social support (FSS) has been shown to be positively associated with better cognitive function, especially memory, in cross-sectional and longitudinal studies. To better understand this complex association, researchers should consider the impact of additional factors that affect both FSS and memory. Therefore, we conducted a systematic review to examine whether one such factor, marital status or related variables (e.g., FSS from spouses compared to FSS from relatives or friends), affects (e.g., confounds or modifies) the association between FSS and memory in middle-aged and older adults.

**Methods:**

We searched PubMed, PsycINFO, and Scopus from database inception to June 2022. Eligible articles examined the association between FSS and memory, and included marital status or related variables in the analysis. Data were synthesized narratively and reported in accordance with the Synthesis without meta-analysis (SWiM) guidelines; risk of bias was assessed using the Newcastle-Ottawa Scale (NOS).

**Results:**

Four articles were included in the narrative synthesis. All four articles had a low risk of bias. Overall findings suggested some positive associations between FSS from a spouse/partner and memory; however, effect sizes were small and similar to other sources of support, including children, relatives, and friends.

**Conclusions:**

Our review is the first attempt to synthesize the literature on this topic. Despite theoretical support for examining the impact of marital status or related variables on the association between FSS and memory, published studies explored this issue secondarily to other research questions.

**Supplementary Information:**

The online version contains supplementary material available at 10.1186/s12877-023-03982-3.

## Background

Memory, one of the six domains of cognitive function, is the capacity to encode, store, and retrieve information [1]. Changes in the memory domain are commonly linked to the aging process, with approximately 40 to 47% of older adults experiencing memory impairment over the age of 65 years [2]. Age-related declines in memory present daily challenges for older adults, including difficulty remembering dates, appointments, and locations. Declines in memory are also strong markers for the incidence of minor and major neurocognitive disorder. Therefore, investigating potentially modifiable risk or protective factors for memory is crucial to help offset future health challenges and promote healthy aging in middle-aged and older adults [3]. One such modifiable risk factor is social support.

Social support broadly refers to the availability of a myriad of social resources that individuals can use to help with decision making, problem solving, and maintaining positive experiences in life [4]. Functional social support (FSS) is one’s perception of whether members of their social network will be available to provide practical help and emotional support when needed, and the satisfaction derived from such help and support [5]. Many researchers believe a focus on FSS is necessary because it relates to the actual amount of help that one believes will be available to meet their needs. Structural support, on the other hand, is an objective count of social relations and activities; higher levels of structural support do not necessarily translate into more help with unmet physical or emotional needs in aging populations.

FSS has been shown to be associated with higher levels of cognitive function, specifically memory, in many cross-sectional and longitudinal studies [6–10]. However, a paucity of literature has examined how this association may differ according to one’s marital status.

Marital status has been associated with differences in memory performance. Specifically, married people report better performances in memory compared to both single and widowed individuals [11–13]. This phenomenon may be explained by a concept known as the “use-it-or-lose-it’ theory [14], which suggests that cognitive stimulation from a partner strengthens mental processes like memory, allowing for more efficient use of neural networks and slowing down age-related declines in memory. Additionally, single and widowed people often have reduced access to individuals who can help with unmet needs (FSS), especially in older age. For widowed individuals, the death of a spouse often signifies a major loss of emotional support (a component of FSS), especially in older adults who may require such support the most. The loss of a loved one can produce major stress and depressive symptomology, both of which have been linked to poor memory performance. Overall, married people or persons in common-law relationships may derive greater benefits from positive associations between FSS and memory, compared to those whose marital status is something other than married or living in common law relationships [12].

Earlier systematic reviews have examined the separate effects of FSS on cognitive functioning or marital status on cognitive functioning [3, 15–18]; however, these reviews did not examine all three variables together. Additionally, none of these reviews were undertaken to address whether marital status or a related variable (e.g., FSS from spouses compared to FSS from relatives or friends – deemed “marital-related variables”) affected (e.g., confounded or modified) the association between FSS and memory in middle-aged or older adults.

We conducted a systematic review to examine whether marital status or marital-related variables affect the association between FSS and memory in middle-aged and older adults. We focused on the memory domain of cognitive function because it is central to healthy aging [19]. Further, memory is associated with everyday physical, behavioural, and social functioning; poor memory is a marker for abnormal aging and major neurocognitive disorders (e.g., Alzheimer’s disease) [20].

## Methods

This review was reported in accordance with the Preferred Reporting Items for Systematic Reviews and Meta-analyses (PRISMA) guidelines [21]. Our protocol was registered in the International Prospective Register of Systematic Reviews (PROSPERO) (registration number: CRD42022352592).

### Eligibility criteria

Articles that met the following eligibility criteria were included in the review:

#### Types of studies

Cohort, case-control, and cross-sectional studies of any language, publication date, and setting, published in scholarly or academic journals. Randomized controlled trials were not considered for inclusion since the nature of the exposure variable (FSS) prohibits randomizing interventions.

#### Types of participants

Adults aged 45 years or over, recruited from any setting (e.g., hospital, community-dwelling, long-term care facility).

#### Types of exposure

The main exposure variable had to be FSS, as defined in the Background section above. Specifically, reviewers included articles that examined the quality of social relationships, such as the ability to provide emotional or informational support and companionship when needed. Included articles needed to examine FSS separately from other social engagement variables. Therefore, studies that examined structural support, defined as quantitative/objective aspects of social relationships, such as the size of one’s social network or the frequency of contact with social network members, were excluded. Furthermore, studies that combined structural and functional social support into one measure did not qualify for the current systematic review. In the absence of a gold standard measure of FSS, we accepted any means of measuring the construct, including validated instruments or single questions asking about levels of support on a Likert scale.

#### Types of outcomes

Memory was the main outcome variable. Articles could investigate any form of memory, e.g., episodic memory, semantic memory, implicit memory, or working memory, provided the memory outcome was reported separately from other cognitive outcomes. Memory had to be measured using a validated scale designed specifically to measure memory. Studies combining memory and other cognitive domains into a global cognitive function index, and only reporting this index, were excluded from the review.

Included articles must have examined the impact of marital status or a marital-related variable on the association between FSS and memory. Examples of ‘impact’ included testing a variable as a covariate, confounder, or effect modifier in regression models examining the relation between FSS and memory. We also included articles that compared associations between FSS from different sources, such as spouses and children, and memory. We included marital-related variables in addition to ‘marital status’ to reflect the fact that multiple measures of one’s marital situation are used in the literature. We could potentially have omitted relevant articles had we restricted our operationalization of marital status to one measure (‘marital status’ alone).

### Search strategy

We searched PubMed, PsycINFO (accessed through APA PsycNET), and Scopus from the inception of each database to June 29th, 2022. A search strategy in PubMed was created with the help of a medical librarian using Medical Subject Headings from the National Library of Medicine. The first author modified the PubMed search syntax to meet the parameters of the other two databases. No restrictions were applied to any of the searches. Search strategies for each database are shown in Additional File 1: Appendix A. The reference lists of included articles were also hand-searched to find potentially relevant articles that were not captured by the search strategy.

### Study selection

All retrieved articles were transferred into Covidence [22] for duplicate identification/removal and screening. The first level of screening was title and abstract. Articles that passed this initial level of screening underwent full-text screening. Two independent reviewers screened each article at both levels using the following four questions, which were based on the eligibility criteria described above: (1) Does this article examine the association between FSS and memory specifically?; (2) Does this article examine a study population of middle-aged and/or older adults (45 years or over)?; (3) Does this article describe a primary or secondary analysis of data and, if so, does the study include a comparison group?; and (4) Does this article examine the role of a marital-related variable in the association between FSS and memory? During title and abstract screening, if reviewers answered “yes” to all four questions or they had inadequate information to assess one or more of the four questions, but they did not answer “no” to any question, then the article was promoted to full-text screening. At the full-text screening level, all four questions required “yes” responses to be included in the review. Disagreements between reviewers were settled by consensus.

### Data extraction process

A data extraction form was created with the following headings: authors, year of publication, nationality of sample population, study design, sample size, percentage of female participants in the sample, exposure variables measured (FSS, marital status), outcome variables measured (memory), covariates, means of employing marital-related variables in the analyses, and a summary of relevant findings. Two reviewers independently extracted data from each article using the same data extraction form and compared their responses to create a comprehensive spreadsheet with information from all included studies. Discrepancies were settled by consensus.

### Data synthesis

The results of the included articles were narratively synthesized and reported in accordance with the Synthesis without meta-analysis (SWiM) guidelines [23] (Additional File 1: Appendix B). SWiM is designed to encourage transparency in the reporting of systematic reviews that employ narrative synthesis and do not contain meta-analysis [23]. In this systematic review, a meta-analysis could not be performed due to the substantial heterogeneity in the methodologies observed in the included articles, especially regarding the use of marital-related variables in analyses, differences in the measures of memory that were employed in these articles, and differences in statistical approaches used to analyze the data. All commentary regarding heterogeneity was based on informal comparisons of between-article differences in areas such as definitions/measures of exposure, measures of marital status or marital-related variables, measures of outcome, and covariates used in regression models.

When possible, we summarized quantitative article results using the regression coefficient ($\widehat{\beta }$). In the event authors of included articles did not report $\widehat{\beta }$, we summarized results with the outcome measures used in the articles (e.g., correlation coefficients). For synthesis purposes, we discussed cross-sectional and longitudinal results separately.

### Risk of bias assessment

To evaluate risk of bias in the included articles, we used the Newcastle-Ottawa Scale (NOS), which involved assessing articles in three domains and awarding points to reflect the degree to which each domain was free of bias [24]. A maximum of 9 points was awarded to each study. All included articles were categorized as high risk of bias (3 or fewer points), moderate risk (4–6 points), or low risk (7–9 points). Two reviewers independently evaluated risk of bias for each article and resolved disagreements by consensus.

## Results

**Fig. 1:**
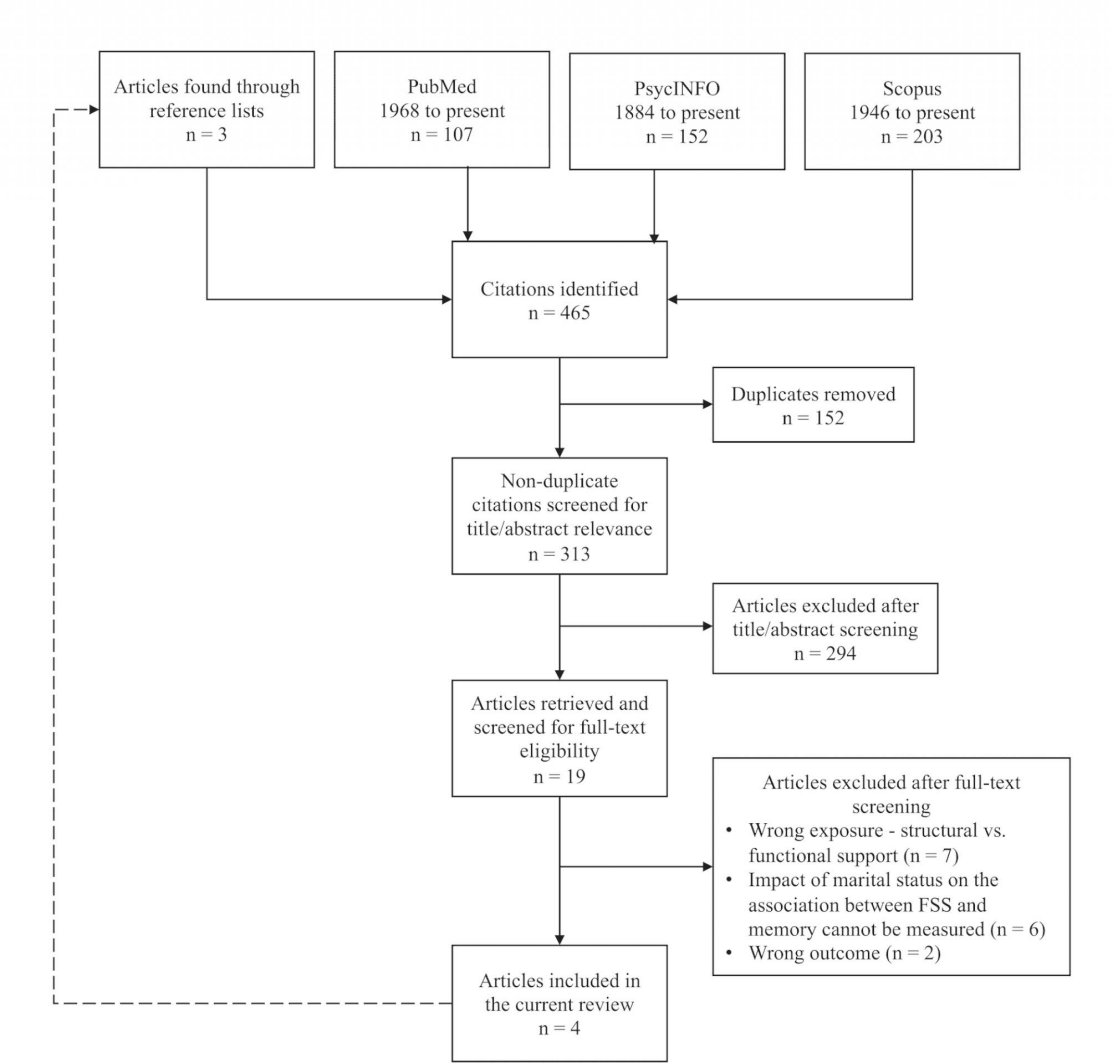
Study flow

The literature search produced 465 records. Following duplicate removal (n = 152), 313 articles were screened at the title and abstract level, and 19 progressed to full-text screening (Fig. 1). Full-text screening led to the exclusion of 15 articles primarily because they measured structural rather than functional aspects of social support. This left four articles for inclusion in the narrative synthesis [25–28]. A list of all excluded articles and their reason for exclusion are available from the authors upon request.

All four articles employed a mix of cross-sectional and longitudinal secondary analyses from three large panel studies, namely the Health and Retirement Study (HRS) from the United States [29], the English Longitudinal Study of Ageing from England (ELSA) [30], and the PATH Through Life Study from Australia [31]. Sample sizes ranged from 1618 [28] to 10 390 [27]. A summary of findings from the included articles can be found in Table 1.

**Table 1:** Data extraction of included articles

Author, Year *Country*	Study Design, *Funding*	Sample Size; %_female_	Exposure(s) measured	Outcome(s) measured	Covariates	Use of marital status	Summary of findings
Liao & Scholes, 2017 *England*	Longitudinal *Funding: Public*	10 241 53.3%	*Positive social support*: measured with three items asking about how much members of one’s social network understand the way they feel, can be relied on, and can open up to them.	*Memory*: measured with time orientation, verbal learning (immediate and delayed recall), and prospective memory tasks.	Age, sex, socioeconomic status (education and wealth), health factors, mobility limitations, depressive symptoms	Modifier	For men, higher between-persons positive social support from a spouse/partner was associated with slower memory decline. For women, higher between-persons positive social support from a spouse/partner was associated with lower baseline memory.
Scholes & Liao, 2022 *England*	Longitudinal *Funding: Public*	10 109 53%	*Social support*: measured with three items asking how much members of one’s social network understand the way they feel, can be relied on, and can open up to them – completed separately for spouse/partner, children, other family, and friends.	*Verbal memory*: measured using immediate and delayed word recall tasks.	Age, wealth, education, smoking and alcohol consumption, physical activity, social participation, physical functioning, depressive symptoms	Modifier	No significant associations were found between spousal support and baseline memory or its rate of change.
Windsor et al., 2014 *Australia*	Longitudinal *Funding: Public & Private*	1618 49.3%	*Positive social exchanges*: measured with five items asking about emotional closeness and spouse dependability.	*Episodic memory*: measured using the California Verbal Learning Test. *Working memory*: measured using the digits backward subtest of the WMS.	Age, sex, education, physical functioning, depressive symptoms	Modifier	No significant associations were found between positive spouse exchanges and episodic or working memory.
Zahodne et al., 2019 *USA*	Longitudinal *Funding: Public*	10 390 59.68%	*Marital status*: dichotomous (married/partnered vs. not married/partnered). *Social support*: measured with three items asking how much members of one’s social network understand the way they feel, can be relied on, and can open up to them – completed separately for spouse, children, family, and friends.	*Episodic memory*: measured with a variant of the CERAD list learning task that assesses immediate and delayed recall.	Age, gender, race/ethnicity, education, baseline assessment wave, depression, physical health/chronic health conditions, and self- rated health	Exposure Modifier	Being partnered and having less social support were each independently associated with higher initial memory. Having a partner was also associated with slower memory decline. Spousal support was not significantly associated with initial memory or subsequent memory change.

*Note.* CERAD = Consortium to Establish a Registry for Alzheimer’s Disease; USA = United States of America; WMS = Wechsler Memory Scale

Three articles had a predominantly female sample. Windsor et al.’s sample [28] was approximately evenly split between male and female participants. The average age of participants in these studies ranged from 62.5 [28] to 68.6 years [27]. The most common covariates adjusted for in the included articles were age, sex, education, physical functioning, and depressive symptoms. Scholes and Liao [25] adjusted for the largest number of covariates (n = 9) and Windsor et al. [28] adjusted for the least number of covariates (n = 5).

FSS was measured in a similar fashion across three of the included articles. Liao and Scholes [26], Scholes and Liao [25], and Zahodne et al. [27] measured FSS with three items asking participants to indicate the extent to which members of their social networks understood their feelings, could be relied upon to help in times of need, and would be available to serve as good listeners [32]. Windsor et al. [28] measured FSS as ‘positive social exchanges’, with five items asking about participants’ emotional closeness and dependability on their spouses.

In the panel studies used by Liao and Scholes [26], Scholes and Liao [25], and Zahodne et al. [27], participants provided four different sets of answers to the three FSS items, one set for each of the following sources of social support: spouses, children, family, and friends. Zahodne et al. [27] excluded individuals from analyses if these individuals did not report the presence of spouses, children, extended family, or friends in their social support networks. In contrast, Liao and Scholes [26] and Scholes and Liao [25] included such individuals in their analyses but assigned them scores of zero on the relevant measures of social support.

Each included article measured memory differently, both in terms of the type of memory as well as the instrument used to collect data. Liao and Scholes [26] measured memory using time orientation, immediate and delayed recall, and prospective memory tasks. Scholes and Liao [25] measured verbal memory using immediate and delayed recall. Windsor et al. [28] used episodic and working memory as their outcomes of interest and assessed episodic memory using an immediate recall task from the California Verbal Learning Test [33]; they assessed working memory with the digits backward subtest of the Wechsler Memory Scale [34]. Zahodne et al. [27] measured episodic memory with the Consortium to Establish a Registry for Alzheimer’s Disease (CERAD) list learning task [35].

### Cross-sectional findings

All four included articles examined the role of source of support as a modifying variable in the association between FSS and memory at baseline. More specifically, authors compared responses on the FSS scale described above for spouses as the source of support to responses for children, family members, and friends as the sources of support.

Windsor et al. [28] reported positive correlations between spousal support and both episodic (*r* = 0.18) and working memory (*r* = 0.04). Similarly, positive correlations were also found for support from friends (episodic memory: *r* = 0.10; working memory: *r* = 0.06) and family (episodic memory: *r* = 0.03; working memory: *r* = 0.07). However, only the correlation between support from friends and episodic memory was statistically significant.

Scholes and Liao [25] reported positive associations between spousal support and verbal memory in men ($\widehat{\beta }$*=* 0.065; *95% CI* = -0.029, 0.160), but negative associations in women ($\widehat{\beta }$*= -*0.043; *95% CI* = -0.128, 0.041). Positive associations were also found for support from children (men: $\widehat{\beta }$*=* 0.078; *95% CI* = -0.013, 0.169; women: $\widehat{\beta }$*=* 0.052; *95% CI* = -0.028, 0.133), and friends (men: $\widehat{\beta }$*=* 0.173; *95% CI* = 0.048, 0.299; women: $\widehat{\beta }$*=* 0.291; *95% CI* = 0.168, 0.414); only the model including support from friends was statistically significant. Conversely, negative associations were found between support from family and verbal memory (men: $\widehat{\beta }$*= -*0.096; *95% CI* = -0.209, 0.017; women: ($\widehat{\beta }$*= -*0.025; *95% CI* = -0.124, 0.075).

Zahodne et al. [27] found spousal support to be positively associated with episodic memory ($\widehat{\beta }$*=* 0.02; *95% CI* = 0.00, 0.05) when compared to support from children ($\widehat{\beta }$*=* 0.00; *95% CI* = -0.02, 0.03), family ($\widehat{\beta }$*= -*0.03; *95% CI* = -0.05, -0.01), and friends ($\widehat{\beta }$*=* 0.00; *95% CI* = -0.02, 0.02). However, only the model including support from family was statistically significant.

Liao and Scholes [26] reported that for women, higher between-person social support from a spouse/partner was significantly associated with lower baseline memory ($\widehat{\beta }$*=* -0.063; *95% CI* = -0.094, -0.031). Similar negative associations were also found for support from children in women ($\widehat{\beta }$*= -*0.006; *95% CI* = -0.040, 0.027), and support from family members (men: $\widehat{\beta }$*=* -0.050; *95% CI* = -0.090, -0.010; women: $\widehat{\beta }$*= -*0.018; *95% CI* = -0.054, 0.019); only the model including support from family in men was statistically significant. Conversely, support from friends (men: $\widehat{\beta }$*=* 0.063; *95% CI* = -0.094, -0.031; women: $\widehat{\beta }$*=* 0.119; *95% CI* = 0.073, 0.164), support from children in men ($\widehat{\beta }$*=* 0.033; *95% CI* = -0.002, 0.068), and support from spouses in men ($\widehat{\beta }$*=* 0.015; *95% CI* = -0.020, 0.049) were positively associated with baseline memory, with support from friends in women being statistically significant.

### Longitudinal findings

All four articles extended the cross-sectional analyses to include multiple time points for the purpose of examining changes in memory over time. Upon following individuals on three occasions over an 8-year interval, Windsor et al. [28] did not find any significant associations between spousal support and changes in both episodic and working memory. A similar pattern was also reported for support from family and longitudinal changes in both episodic and working memory.

Scholes and Liao [25] reported positive associations between spousal support and change in verbal memory during a 16-year follow-up for both men ($\widehat{\beta }$ = 0.016; *95% CI* = -0.003, 0.035) and women ($\widehat{\beta }$ = 0.003; *95% CI* = -0.014, 0.020). Similarly, positive associations were also found for support from children (men: $\widehat{\beta }$ = 0.020; *95% CI* = 0.002, 0.039; women: $\widehat{\beta }$ = 0.000; *95% CI* = -0.017, 0.018), family members (men: $\widehat{\beta }$ = 0.006; *95% CI* = -0.015, 0.028; women: $\widehat{\beta }$ = 0.012; *95% CI* = -0.009, 0.033), and friends (men: $\widehat{\beta }$ = 0.006; *95% CI* = -0.020, 0.033; women: $\widehat{\beta }$ = 0.003; *95% CI* = -0.025, 0.030).

Conversely, Zahodne et al. [27] reported negative associations between spousal support and change in episodic memory during a 6-year follow-up ($\widehat{\beta }$ = -0.02; *95% CI* = -0.10, 0.07), when compared to support from children ($\widehat{\beta }$ = 0.00; *95% CI* = -0.07, 0.07), and family members ($\widehat{\beta }$ = 0.03; *95% CI* = -0.04, 0.09). Support from friends was also negatively associated with longitudinal change in memory ($\widehat{\beta }$ = -0.06; *95% CI* = -0.12, 0.00).

When stratifying by sex, Liao and Scholes [26] reported that higher between-person social support from a spouse/partner was significantly associated with slower memory decline over an 8-year period for both men and women (men: $\widehat{\beta }$ = 0.006; *95% CI* = 0.000, 0.012; women: $\widehat{\beta }$ = 0.009; *95% CI* = 0.004, 0.015). Similar positive associations were also found between memory decline and support from children (men: $\widehat{\beta }$ = 0.004; *95% CI* = -0.002, 0.009; women: $\widehat{\beta }$ = 0.005; *95% CI* = -0.001, 0.010), family members (men: $\widehat{\beta }$ = 0.004; *95% CI* = -0.003, 0.011; women: $\widehat{\beta }$ = 0.007; *95% CI* = 0.00, 0.013), and friends (men: $\widehat{\beta }$ = 0.001; *95% CI* = -0.007, 0.009; women: $\widehat{\beta }$ = 0.006; *95% CI* = -0.002, 0.014).

### Risk of bias

A summary of the risk of bias assessments is described in Additional File 1: Appendix C. Overall, all four articles had a low risk of bias. Sources of bias were mainly concentrated in the outcome section of the NOS because most studies either did not report the follow-up rate or did not provide descriptions regarding participants lost to follow-up. We noted that none of the included articles provided sample size calculations.

## Discussion

This narrative synthesis described the impact of marital-related variables on the association between FSS and memory. Across the included articles, the findings suggested some positive associations between FSS from a spouse/partner and memory, notably when the results were stratified by sex. However, the effect sizes were small and similar to other sources of support, including children, relatives, and friends. The small effect sizes did not allow us to ascertain whether spousal support was more effective than these other sources of support. The matter was complicated by the lack of statistical significance for many results, likely due to underpowered analyses, which further inhibited our ability to draw conclusions about the strength and direction of effects. Further, heterogeneity in exposure and outcome measurement, as well as different sets of covariates employed in the analyses, diminished the comparability of results across articles. Nonetheless, the small effect sizes observed in our work were consistent with the findings reported in some primary studies [9] and in previous systematic reviews of social relationships and cognitive function [17].

Some of our review’s findings are consistent with a growing body of literature showing that marital partners may act as a protective buffer against cognitive decline [11, 12, 36]. The ‘use it or lose it’ theory emphasizes that engaging in social interactions with specific members of one’s social network – especially in the frequent and regularized fashion of spousal relationships – stimulates areas of the brain related to cognitive processes such as memory [14, 37]. These cognitive demands build up a reserve capacity allowing married or partnered individuals to utilize neural networks more efficiently, thus reducing the effects of cognitive decline [37] relative to single, widowed, divorced or separated individuals [36].

Previous systematic reviews have also shown that the relationship between social support and cognitive factors may vary depending on one’s personal characteristics [15]. It is possible that, according to the use-it-or-lose-it theory, marital status may alter the magnitude and direction of the relationship between FSS and memory. Future research would benefit from longitudinal comparisons of FSS and memory across different strata of marital status to test this theory. Although many reported results from the included studies were not statistically significant, these null findings did not necessarily suggest the absence of an association between social support from a spouse/partner and memory, given the possibility of underpowered analyses. Furthermore, when stratifying their findings by sex, Liao and Scholes [26] found that higher FSS from a spouse/partner was associated with lower baseline memory specifically in women. This suggests sex is an important variable in this relationship, as previous research has shown that women exchange social support with a wider range of social partners and are more sensitive to appraisals of partnership quality than men [38, 39]. Therefore, the wider social networks to which women belong may make marital status appear stronger for women, compared to men. These findings further emphasize the importance of context when examining the association between social variables and cognitive function, and suggest that any understanding of this relationship should include sex as a variable of interest.

Furthermore, most of the articles included in the current review dichotomized marital status into married/partnered versus unmarried/unpartnered individuals. In these instances, the unmarried group encompassed all individuals who were single, never married, divorced, separated, or widowed. However, such categorization may have obscured important inter-group differences because widowed, divorced, or separated individuals may share very diverse experiences from those who were never married due to the grief and stress associated with losing a loved one or the dissolution of a long-term relationship.

Overall, some findings suggested spousal support had a positive impact on memory, while in other results this impact was no stronger than the effect of social support from friends, relatives, and children. These mixed findings might be explained by study features that differed across the articles, such as measures of memory, design factors (e.g., length of follow-up), sample sizes, and covariates.

Several other factors detracted from our ability to draw consistent conclusions across the reported results. Firstly, many findings were statistically nonsignificant and we could not ascertain whether this reflected low power or no actual associations between the variables of interest. Secondly, we could not assess whether effect modification based on marital status or marital-related variables existed because none of the articles explored this question. Thirdly, the pool of included articles was not sufficiently large enough to investigate whether FSS and marital status or marital-related variables had different impacts on various types of memory.

### Strengths and limitations

The current systematic review is the first to summarize and appraise research about the effect of marital status or related variables on the association between FSS and memory. Previous reviews focused on associations between either social support or marital relationships and the outcomes of global cognitive function or dementia. These reviews did not include articles exploring associations between FSS, marital status or a related variable, and memory within the same analyses. Unlike these earlier reviews, we sought articles that examined the effect of marital-related variables on the association between FSS and memory. In addition, we performed a comprehensive literature search and thorough narrative assessment using the SWiM guidelines. We also searched the reference lists/bibliographies of included studies to identify articles that met our eligibility criteria but were not detected in the literature search. Last, we used the AMSTAR 2 checklist [40] to appraise our review and scored 13 out of 13 points (omitting three questions pertaining to meta-analysis) (see Additional File 1: Appendix D).

To promote consistency between reviewers in terms of screening, risk of bias assessment, and data extraction, a guidance document on the eligibility criteria, including formal definitions of FSS and memory, was provided to all reviewers. Regular meetings were also held throughout the review process to answer any questions and make sure reviewers had a good understanding of the study objectives.

Although this review had many strengths, we could not perform a meta-analysis due to the substantial clinical heterogeneity in the methodologies observed in the included articles, especially regarding the definition and use of marital status in analyses. The heterogeneity in definitions and measures of memory also precluded meta-analysis. Memory measures included the Wechsler Memory Scale [34], California Verbal Learning Test [33], and the CERAD list learning task [35].

### Future implications

The current review identified several gaps in the literature. For instance, among the 465 citations identified through our search, only the four included publications assessed a martial-related variable that was intertwined with FSS and memory. We came across other articles that included marital status, FSS and memory in the same analyses [5, 7, 8], but these articles were not included in the current review because the authors utilized FSS and marital status as separate independent variables in different multivariable regression models, rather than examining whether marital status impacted the association between FSS and memory as an effect modifier (perhaps through stratification) or confounder (by running models with and without marital status and comparing Δ$\widehat{\beta }$ for FSS across models).

Of the included articles, two studies examined married/partnered and single individuals simultaneously within their analyses of FSS and memory [25, 26]. However, relationship type (i.e., spouse, children, other family, and friends) rather than marital status acted as the modifier within the analyses of these studies. Within these analyses, individuals who were unmarried (i.e., single, divorced, separated, or widowed) were often given a score of zero on indices of social support. However, it is possible that, in some cases, single individuals may derive a greater quality of support from close friends or family than married individuals from their spouse or partners. Thus, future studies should examine how marital status, specifically, may act as an effect modifier of the association between FSS and memory, rather than relationship type.

Furthermore, the included articles did not examine the possibility of a bidirectional relationship between FSS and memory. Lower quality of social support within close relationships may deprive individuals of cognitive stimuli and subsequently lead to poor late-life memory; however, memory impairment may also lead to a low quality of social support. To examine this potential bi-directionality, future studies should employ longitudinal study designs with longer follow-up periods. Moreover, informed by the findings of Liao and Scholes [26], future studies may benefit from exploring the associations between marital status, FSS, and memory stratified by sex, or other biopsychosocial determinants of health such as age.

## Conclusions

The current systematic review suggests that marital-related variables may affect the association between social support and memory in middle-aged and older adults, although the evidence is equivocal. Also, a large number of factors are intertwined with marital status and social support, such as marital quality and length of relationship, which were not considered in the included studies. Future research undertaken to examine the effect of marital status or marital-related variables on the association between FSS and memory should consider these additional contextual factors. Researchers also need to be consistent in definitions and operationalizations of social support and memory to permit the creation of a uniform evidence base.

## Electronic supplementary material

Below is the link to the electronic supplementary material.

Additional File 1

## Data Availability

All data generated or analysed during this study are included in this published article (and its supplementary information file).

## List of Abbreviations

AMSTAR: Assessment of multiple systematic reviews
CERAD: Consortium to Establish a Registry for Alzheimer’s Disease
ELSA: English Longitudinal Study of Ageing
FSS: Functional social support
HRS: Health and Retirement Study
NOS: Newcastle-Ottawa Scale
PRISMA: Preferred Reporting Items for Systematic reviews and Meta-Analyses
PROSPERO: International Prospective Register of Systematic Reviews
SWiM: Synthesis without meta-analysis.

## References

[1] Craik FIM, Rose NS (2012). Memory encoding and aging: a neurocognitive perspective. Neurosci Biobehav Rev.

[2] Aigbogun MS, Stellhorn R, Krasa H, Kostic D (2017). Severity of memory impairment in the elderly: Association with health care resource use and functional limitations in the United States. Alzheimer’s and Dementia: Diagnosis Assessment and Disease Monitoring.

[3] Kelly ME, Duff H, Kelly S, McHugh Power JE, Brennan S, Lawlor BA (2017). The impact of social activities, social networks, social support and social relationships on the cognitive functioning of healthy older adults: a systematic review. Syst Rev.

[4] Benca-Bachman CE, Najera DD, Whitfield KE, Taylor JL, Thorpe RJ, Palmer RHC (2020). Quality and quantity of social support show differential associations with stress and depression in African Americans. Am J Geriatric Psychiatry.

[5] Gow AJ, Corley J, Starr JM, Deary IJ (2013). Which social network or support factors are associated with cognitive abilities in old age?. Gerontology.

[6] Hughes TF, Andel R, Small BJ, Borenstein AR, Mortimer JA. The association between social resources and cognitive change in older adults: Evidence from the Charlotte County Healthy Aging Study. J Gerontol B Psychol Sci Soc Sci [Internet]. 2008;63(4):P241–4. Available from: https://academic.oup.com/psychsocgerontology/article/63/4/P241/581727. 10.1093/geronb/63.4.p241 18689766

[7] Hülür G (2022). Structural and functional aspects of social relationships and episodic memory: between-person and within-person associations in middle-aged and older adults. Gerontology.

[8] Ge S, Wu B, Bailey DE, Dong XQ. Social support, social strain, and cognitive function among community-dwelling U.S. Chinese older adults. Journals of Gerontology - Series A Biological Sciences and Medical Sciences. 2017;72(S1):S16–21. 10.1093/gerona/glw221 PMC5861953 28575260

[9] Ohman A, Maxwell CJ, Tyas SL, Oremus M. Subtypes of social support availability are not differentially associated with memory: a cross-sectional analysis of the Comprehensive Cohort of the Canadian Longitudinal Study on Aging. Aging, Neuropsychology, and Cognition. 2022;1–16. 10.1080/13825585.2022.2030294 35086434

[10] Seeman TE, Miller-Martinez DM, Stein Merkin S, Lachman ME, Tun PA, Karlamangla AS (2011). Histories of social engagement and adult cognition: midlife in the U.S. study. J Gerontol B Psychol Sci Soc Sci.

[11] Liu H, Zhang Y, Burgard SA, Needham BL (2019). Marital status and cognitive impairment in the United States: evidence from the National Health and Aging Trends Study. Ann Epidemiol.

[12] Mousavi-Nasab SMH, Kormi-Nouri R, Sundström A, Nilsson LG (2012). The effects of marital status on episodic and semantic memory in healthy middle-aged and old individuals. Scand J Psychol.

[13] Aartsen MJ, van Tilburg T, Smits CHM, Comijs HC, Knipscheer KCPM (2005). Does widowhood affect memory performance of older persons?. Psychol Med.

[14] Hultsch DF, Hertzog C, Small BJ, Dixon RA (1999). Use it or lose it: engaged lifestyle as a buffer of cognitive decline in aging?. Psychol Aging.

[15] Costa-Cordella S, Arevalo-Romero C, Parada FJ, Rossi A (2021). Social support and cognition: a systematic review. Front Psychol.

[16] Sommerlad A, Ruegger J, Singh-Manoux A, Lewis G, Livingston G (2018). Marriage and risk of dementia: systematic review and meta-analysis of observational studies. J Neurol Neurosurg Psychiatry.

[17] Kuiper JS, Zuidersma M, Zuidema SU, Burgerhof JGM, Stolk RP, Oude Voshaar RC (2016). Social relationships and cognitive decline: a systematic review and meta-analysis of longitudinal cohort studies. Int J Epidemiol.

[18] Piolatto M, Bianchi F, Rota M, Marengoni A, Akbaritabar A, Squazzoni F. The effect of social relationships on cognitive decline in older adults: an updated systematic review and meta-analysis of longitudinal cohort studies. BMC Public Health. 2022 Feb;11(1):278. 10.1186/s12889-022-12567-5 PMC8831686 35148704

[19] Umberson D, Montez JK (2010). Social relationships and health: a flashpoint for health policy. J Health Soc Behav.

[20] Tuokko H, Griffith LE, Simard M, Taler V (2017). Cognitive measures in the canadian longitudinal study on aging. Clin Neuropsychol.

[21] Page MJ, McKenzie JE, Bossuyt PM, Boutron I, Hoffmann TC, Mulrow CD (2021). Updating guidance for reporting systematic reviews: development of the PRISMA 2020 statement. J Clin Epidemiol.

[22] Covidence systematic review software [Internet]. Veritas Health Innovation.Melbourne, Australia; Available from: www.covidence.org.

[23] Campbell M, McKenzie JE, Sowden A, Katikireddi SV, Brennan SE, Ellis S (2020). Synthesis without meta-analysis (SWiM) in systematic reviews: reporting guideline. BMJ.

[24] Wells G, Shea B, O’Connell D, Peterson J, Welch V, Losos M et al. The Newcastle-Ottawa Scale (NOS) for assessing the quality of nonrandomised studies in meta-analyses [Internet]. Ottawa Hospital Research Institute. 2009. Available from: https://www.ohri.ca/programs/clinical_epidemiology/oxford.asp.

[25] Scholes S, Liao J. Social support, social strain and declines in verbal memory: Sex-specific associations based on 16-year follow-up of the English Longitudinal Study of Ageing cohort. Aging Ment Health. 2022;1–9. 10.1080/13607863.2022.2089628 35735097

[26] Liao J, Scholes S (2017). Association of social support and cognitive aging modified by sex and relationship type: a prospective investigation in the English Longitudinal Study of Ageing. Am J Epidemiol.

[27] Zahodne LB, Ajrouch KJ, Sharifian N, Antonucci TC (2019). Social relations and age-related change in memory. Psychol Aging.

[28] Windsor TD, Gerstorf D, Pearson E, Ryan LH, Anstey KJ (2014). Positive and negative social exchanges and cognitive aging in young-old adults: Differential associations across family, friend, and spouse domains. Psychol Aging.

[29] Sonnega A, Weir DR (2014). The Health and Retirement Study: a public data resource for research on aging. Open Health Data.

[30] Steptoe A, Breeze E, Banks J, Nazroo J (2013). Cohort profile: the English Longitudinal Study of Ageing. Int J Epidemiol.

[31] Anstey KJ, Christensen H, Butterworth P, Easteal S, Mackinnon A, Jacomb T (2012). Cohort profile: the PATH through life project. Int J Epidemiol.

[32] Schuster TL, Kessler RC, Aseltine RH (1990). Supportive interactions, negative interactions, and depressed mood. Am J Community Psychol.

[33] Delis DC, Kramer JH, Kaplan E, Ober BA (1987). California Verbal Learning Test: adult version manual.

[34] Wechsler D (1945). A standardized memory scale for clinical use. J Psychol.

[35] Ofstedal MB, Fisher GG, Herzog AR. Documentation of cognitive functioning measures in the Health and Retirement Study [Internet]. Ann Arbor, MI; 2005. Available from: https://hrs.isr.umich.edu/sites/default/files/biblio/dr-006.pdf.

[36] van Gelder BM, Tijhuis M, Kalmijn S, Giampaoli S, Nissinen A, Kromhout D. Marital status and living situation during a 5-year period are associated with a subsequent 10-year cognitive decline in older men: The FINE Study. J Gerontol B Psychol Sci Soc Sci [Internet]. 2006;61(4):P213–9. Available from: https://academic.oup.com/psychsocgerontology/article/61/4/P213/603665. 10.1093/geronb/61.4.p213 16855033

[37] Sörman DE, Rönnlund M, Sundström A, Norberg M, Nilsson LG (2017). Social network size and cognitive functioning in middle-aged adults: cross-sectional and longitudinal associations. J Adult Dev.

[38] Carstensen LL, Gottman JM, Levenson RW (1995). Emotional behavior in long-term marriage. Psychol Aging.

[39] McLaughlin D, Vagenas D, Pachana NA, Begum N, Dobson A (2010). Gender differences in social network size and satisfaction in adults in their 70s. J Health Psychol.

[40] Shea BJ, Reeves BC, Wells G, Thuku M, Hamel C, Moran J (2017). AMSTAR 2: a critical appraisal tool for systematic reviews that include randomised or non-randomised studies of healthcare interventions, or both. BMJ.

