# Supplementary material for: Examining the association between functional social support, marital status, and memory: a systematic review
Source: BMC Geriatr. 2023 May 12;23:290. doi: 10.1186/s12877-023-03982-3 (PMC10182629; doi:10.1186/s12877-023-03982-3)
Supplement: Supplementary file 1 — Additional File 1 [file 12877_2023_3982_MOESM1_ESM.docx]

# Appendix A: Search strategy used for each database

**PSYCInfo:**

Any Field: "social environment" *OR* Any Field: "social isolation" *OR* Any Field: "social support" *OR* Any Field: "social network*" *OR* Any Field: "social relationship*" *OR* Any Field: "social cohesion" *OR* Any Field: "community network*" *OR* Any Field: "social resources" *OR* Any Field: "social participation" *AND* Any Field: memory *OR* Any Field: "immediate recall" *OR* Any Field: "delayed recall" *AND* Any Field: "marital status" *OR* Any Field: marriage *OR* Any Field: married *OR* Any Field: divorced *OR* Any Field: widow* *OR* Any Field: spous* *OR* Any Field: wife *OR* Any Field: husband

**PubMed:**

(social environment[tw] OR social isolation[tw] OR social support*[tw] OR social network*[tw] OR social relationship*[tw] OR social cohesion[tw] OR community network*[tw] OR social resources[tw] OR social participation[tw]) AND (memory[tw] OR working memory[tw] OR memory decline[tw] OR memory loss[tw] OR recall memory[tw] OR immediate recall[tw] OR delayed recall[tw] OR memory function[tw]) AND (marital status[tw] OR marriage[tw] OR married[tw] OR divorced[tw] OR widow*[tw] OR spous*[tw] OR wife[tw] OR husband[tw])

**Scopus:**

( TITLE-ABS-KEY ( "social environment" OR "social isolation" OR "social support" OR "social network*" OR "social relationship*" OR "social cohesion" OR "community network*" OR "social resources" OR "social participation" ) AND TITLE-ABS-KEY ( memory OR "working memory" OR "memory decline" OR "memory loss" OR "recall memory" OR "immediate recall" OR "delayed recall" OR "memory function" ) AND TITLE-ABS-KEY ( "marital status" OR marriage OR married OR divorced OR widow* OR spous* OR wife OR husband ) )

*Note: Rather than searching controlled vocabulary and natural language individually, we opted to use the [tw] term in PubMed and ‘Any Field’ in PsycINFO to capture a broader scope of relevant articles within our search.*

# Appendix B: SWiM reporting guideline

| **SWiM reporting item** | **Item description** | **Page in manuscript where item is reported** | **Other** |
| --- | --- | --- | --- |
| *Methods* | | | |
| **1** Grouping studies for synthesis | 1a) Provide a description of, and rationale for, the groups used in the synthesis (e.g., groupings of populations, interventions, outcomes, study design) | 8-9 |  |
|  | 1b) Detail and provide rationale for any changes made subsequent to the protocol in the groups used in the synthesis | N/A | No deviation from protocol |
| **2** Describe the standardised metric and transformation methods used | Describe the standardised metric for each outcome. Explain why the metric(s) was chosen, and describe any methods used to transform the intervention effects, as reported in the study, to the standardised metric, citing any methodological guidance consulted | 9 |  |
| **3** Describe the synthesis methods | Describe and justify the methods used to synthesise the effects for each outcome when it was not possible to undertake a meta-analysis of effect estimates. | 8-9 |  |
| **4** Criteria used to prioritise results for summary and synthesis | Where applicable, provide the criteria used, with supporting justification, to select the particular studies, or a particular study, for the main synthesis or to draw conclusions from the synthesis (e.g., based on study design, risk of bias assessments, directness in relation to the review question). | 7-8 |  |
| **5** Investigation of heterogeneity in reported effects | State the method(s) used to examine heterogeneity in reported effects when it was not possible to undertake a meta-analysis of effect estimates and its extensions to investigate heterogeneity. | 8-9 |  |
| **6** Certainty of evidence | Describe the methods used to assess certainty of the synthesis findings. | Addressed throughout the manuscript |  |
| **7** Data presentation methods | Describe the graphical and tabular methods used to present the effects (e.g., tables, forest plots, harvest plots).  Specify key study characteristics (e.g., study design, risk of bias) used to order the studies, in the text and any tables or graphs, clearly referencing the studies included. | 37-38 |  |
| *Results* | | | |
| **8** Reporting results | For each comparison and outcome, provide a description of the synthesised findings, and the certainty of the findings. Describe the result in language that is consistent with the question the synthesis addresses, and indicate which studies contribute to the synthesis | 10-14 |  |
| *Discussion* |  |  |  |
| **9** Limitations of the synthesis | Report the limitations of the synthesis methods used and/or the groupings used in the synthesis, and how these affect the conclusions that can be drawn in relation to the original review question | 18-19 |  |

**Appendix C: Newcastle-Ottawa Scale for quality assessment of cross-sectional studies**

| Study | Selection | Comparability | Outcome | Total Score | Risk of bias |
| --- | --- | --- | --- | --- | --- |
| Liao & Scholes, 2017 | **** | ** | ** | 8 | low |
| Scholes & Liao, 2022 | **** | ** | ** | 8 | low |
| Windsor et al., 2014 | **** | ** | *** | 9 | low |
| Zahodne et al., 2019 | *** | ** | ** | 7 | low |

*Notes.* The Newcastle–Ottawa Scale for quality assessment of cohort studies uses a star rating system. Each study can earn a maximum of 9 stars (4 for selection, 2 for comparability, and 3 for outcome). For the comparability section, a study is awarded 1 star if it controls for one of the most important factors (age, sex, or education) or one of the following other factors: marital status, depressive symptoms, income, living arrangement, functional status, chronic health conditions, smoking or alcohol use, and 2 stars for both (Wells et al., 2009). For the outcome section, studies were awarded one star if the outcome was assessed with an independent structured assessment rather than an independent blind assessment, as per the adapted NOS (Moskalewicz & Oremus, 2020).

# Appendix D: AMSTAR-2 Checklist

| **Questions** | | **Overall Decision** |
| --- | --- | --- |
| 1. **Did the research questions and inclusion criteria for the review include the components of PICO?** | | Yes  No |
|  | Population |  |
|  | Intervention |  |
|  | Comparator group |  |
|  | Outcome |  |
|  | Timeframe for follow-up (Optional) |  |
| 1. **Did the report of the review contain an explicit statement that the review methods were established prior to the conduct of the review and did the report justify any significant deviations from the protocol?** | | Yes (if answered ‘yes’ to all seven components)  Partial yes (if answered ‘yes’ to the first four components)  No |
|  | Review question(s) |  |
|  | Search strategy |  |
|  | Inclusion/exclusion criteria |  |
|  | Risk of bias (RoB) assessment |  |
|  | Data synthesis plan |  |
|  | Plan for investigating causes of heterogeneity |  |
|  | Justification for deviations from the protocol |  |
| 1. **Did the review authors explain their selection of the study designs for inclusion in the review?** | | Yes  No |
|  | Explanation for including only randomized controlled studies (RCT) |  |
|  | OR Explanation for including only non-randomized studies (NRSI) |  |
|  | OR Explanation for including both RCTs and NRSI |  |
| 1. **Did the review authors use a comprehensive literature search strategy?** | | Yes (if answered ‘yes’ to all eight components)  Partial yes (if answered ‘yes’ to the first three components)  No |
|  | Searched $\geq$ 2 relevant databases |  |
|  | Provided key words and/or search strategy |  |
|  | Provided justification for publication restrictions (i.e., language) |  |
|  | Searched relevant grey literature |  |
|  | Consulted content experts in the field |  |
|  | Searched the reference lists of included studies |  |
|  | Searched trial/study registries |  |
|  | Conducted search within 24 months of completion of the review |  |
| 1. **Did the review authors perform study selection in duplicate?** | | Yes  No |
|  | At least two reviewers independently agreed on selection of eligible studies and achieved consensus on which studies to include |  |
|  | OR Two reviewers selected a sample of eligible studies and achieved good agreement (at least 80 percent), with the remainder selected by one  reviewer |  |
| 1. **Did the review authors perform data extraction in duplicate?** | | Yes  No |
|  | At least two reviewers achieved consensus on which data to extract from included studies |  |
|  | OR Two reviewers extracted data from a sample of eligible studies and achieved agreement of at least 80% with the remainder extracted by one reviewer |  |
| 1. **Did the review authors provide a list of excluded studies and justify the exclusions?** | | Yes (if answered ‘yes’ to both components)  Partial yes (if answered ‘yes’ to the first component)  No |
|  | Provided a list of all potentially relevant studies that were read in full-text form but excluded from the review |  |
|  | Justified the exclusion from the review of each potentially relevant study |  |
| 1. **Did the review authors describe the included studies in adequate detail?** | | Yes  No |
|  | Described population in detail |  |
|  | Described intervention (including doses where relevant) |  |
|  | Described comparators (including doses where relevant) |  |
|  | Described study’s setting |  |
|  | Described time frame for follow-up |  |
| 1. **Did the review authors use a satisfactory technique for assessing the RoB in individual studies that were included in the review?** | | Yes (if answered ‘yes’ to all four components)  Partial yes (if answered ‘yes’ to the first two components)  No |
|  | For reviews including only NRSI, must have assessed ROB from confounding |  |
|  | AND selection bias |  |
|  | AND methods used to ascertain exposures and outcomes |  |
|  | AND selection of the reported result from among multiple measurements or analyses of a specified outcomes |  |
| 1. **Did the review authors report on the sources of funding for the studies included in the review?** | | Yes  No |
|  | Reported the sources of funding (when available) for individual studies included in the review |  |
| 1. **If meta-analysis was performed did the review authors use appropriate methods for statistical combination of results?** | | Yes  No  No meta-analysis conducted |
|  | For reviews including only NRSI, the authors justified combining the data in a meta-analysis |  |
|  | Used an appropriate weighted technique to combine study results, adjusting for heterogeneity if present |  |
|  | Statistically combined effect estimates from NRSI that were adjusted for confounding, rather than combining raw data, or justified combining raw data when adjusted effect estimates were not available |  |
|  | Reported separate summary estimates for RCTs and NRSI separately when both were included in the review |  |
| 1. **If meta-analysis was performed, did the review authors assess the potential impact of RoB in individual studies on the results of the meta-analysis or other evidence synthesis?** | | Yes  No  No meta-analysis conducted |
|  | Included only low risk of bias RCTs |  |
|  | OR, if the pooled estimate was based on RCTs and/or NRSI at variable RoB, the authors performed analyses to investigate possible impact of RoB on summary estimates of effect. |  |
| 1. **Did the review authors account for RoB in individual studies when interpreting/ discussing the results of the review?** | | Yes  No |
|  | Included only low risk of bias RCTs |  |
|  | OR if RCTs and/or NRSI with moderate to high RoB were included, the review discussed the likely impact of RoB on the results |  |
| 1. **Did the review authors provide a satisfactory explanation for, and discussion of, any heterogeneity observed in the results of the review?** | | Yes  No |
|  | There was no significant heterogeneity in the results |  |
|  | OR if heterogeneity was present, the authors investigated the sources of any heterogeneity in the results and discussed their impact on the results of the review |  |
| 1. **If they performed quantitative synthesis did the review authors carry out an adequate investigation of publication bias (small study bias) and discuss its likely impact on the results of the review?** | | Yes  No  No meta-analysis conducted |
|  | Performed graphical or statistical tests for publication bias and discussed the likelihood and magnitude of impact of publication bias |  |
| 1. **Did the review authors report any potential sources of conflict of interest, including any funding they received for conducting the review?** | | Yes  No |
|  | The authors reported no competing interest |  |
|  | OR The authors described their funding sources and how they managed potential conflicts of interest |  |

*Notes*. 13 out of the 16 questions were applicable to our review (i.e., items related to meta-analysis were not considered). Out of 13 questions, we scored ‘yes’ to 11 items, and ‘partial yes’ to two items.
